# The Effect of Heat Treatment on the Microstructure and Mechanical Properties of P91 Steel for High-Temperature Applications

**DOI:** 10.3390/ma19112281

**Published:** 2026-05-28

**Authors:** Karolina Kowalczyk-Skoczylas, Agata Gawron, Krzysztof Aniołek, Edyta Matyja, Mateusz Skwarski

**Affiliations:** 1 Faculty of Science and Technology, Institute of Materials Engineering, University of Silesia, 75 Pułku Piechoty 1A, 41-500 Chorzów, Poland; krzysztof.aniolek@us.edu.pl (K.A.); edyta.matyja@us.edu.pl (E.M.); 2 Scientific and Technical Services Company “Pro Novum”, Wróbli 38, 40-534 Katowice, Poland; agata-gawron@tlen.pl; 3 Department of Metal Forming Welding and Metrology, Faculty of Mechanical Engineering, Wroclaw University of Science and Technology, Wybrzeże Wyspiańskiego 27, 50-370 Wrocław, Poland; mateusz.skwarski@pwr.edu.pl

**Keywords:** steel, P91 steel, heat treatment, mechanical properties, microstructure, scanning electron microscopy

## Abstract

The effect of high-temperature heat treatment on the microstructure and mechanical properties of post-operation P91 steel (X10CrWMoVNb9-1) was investigated. The tensile test results confirmed the significant effects of heat treatment type and duration on the properties of P91 steel. The absence of tempering resulted in high strength, reduced ductility, and limited microstructural stability. The tensile strength after normalization was nearly twice as high (1330 MPa) than the UTS value of the as-received sample (692 MPa). The decrease in elongation (9.9%) was related to the presence of supersaturated martensite in the microstructure. Tempering at 780 °C for 4 h with air cooling decreased the strength to UTS = 642 MPa and simultaneously increased the ductility (EL = 11.2%) at a hardness of 240 HV0.1. These changes were associated with microstructural evolution, including the formation and dispersion of precipitates. The most uniform microstructure, characterized by a relatively even distribution of 0.30 µm precipitates and reduced martensitic morphology, was observed after double tempering for 2 h + 2 h at 780 °C with air cooling. High-temperature tempering significantly changed the fracture characteristics and fracture morphology, eliminating martensite brittleness while gradually restoring the ductile nature of the fracture. The results indicate that controlled tempering allows for the formation of a stable microstructure with balanced mechanical properties suitable for high-temperature operation.

## 1. Introduction

Energy systems, especially thermal power plants and combined heat and power plants, are increasingly operating under high-temperature and high-pressure conditions, which necessitate the use of advanced construction materials that are resistant to degradation during long-term operation [1,2]. For such applications, heat-resistant steels are particularly important because, in addition to high mechanical strength, they exhibit microstructural stability as well as resistance to creep, oxidation, and corrosion at elevated temperatures [2]. Among the many grades of boiler steels designed for operation under demanding thermal conditions, P91 steel remains one of the most commonly used materials in pressure systems, including steam superheaters and steam pipelines [3]. P91 steel exhibits a balanced combination of high mechanical strength, creep resistance at temperatures up to approximately 600 °C, good weldability, and predictable microstructural stability during long-term operation [4,5]. It is classified as a high-alloy, heat-resistant boiler steel due to its elevated chromium and molybdenum content, along with small amounts of vanadium and niobium [6]. Compared to other boiler steels, it exhibits beneficial anti-corrosion properties, including resistance to steam-induced corrosion. Furthermore, this steel exhibits relatively stable microstructural behavior after high-temperature aging [7].

Despite the passage of time and the emergence of new materials, such as P92 steel [8] and austenitic heat-resistant steels, P91 steel remains competitive due to its wide availability and relatively low production costs. However, proper heat treatment is crucial—especially at the end of service life or as part of material regeneration—as it allows the recovery of tensile strength, ductility, hardness, and a stable tempered martensitic microstructure, thereby limiting the degradation effects that occur during high-temperature operation [9,10]. This paper analyzes the effect of high-temperature heat treatment [11,12,13], particularly tempering, on the structure and mechanical properties of P91 steel in the post-operation state. Such investigations are of practical importance because they enable the assessment of the possibility of the further use of components after the end of their service life or that have been reconditioned [1,2,3]. Analyzing the microstructure and mechanical properties of steel components after heat treatment allows the assessment of whether it is possible to partially restore the features necessary for safe operation in high-temperature conditions [12]. Conducting such studies is important not only from the perspective of material diagnostics but also for evaluating the cost-effectiveness of maintaining power installations.

Research on the effect of heat treatment on the mechanical properties and microstructure of P91 steel has been described in articles by Ch. Pandey [11,12,13] as well as the present authors [14,15]. In a paper [16], the authors analyzed the effect of post-weld heat treatment on steel. To obtain a homogeneous tempered martensitic microstructure and balanced mechanical performance, the material was normalized and tempered below the Ac1 temperature. The changes in the microstructure and mechanical properties of P91 steel [17] after long-term operation for 70,000 h at 560 °C and 27.5 MPa were described in an article by C. Kolan [15]. The microstructure of the steel after long-term operation exhibited only slight degradation. The mechanical properties of steel after operation remained higher than the standard requirements for P91 boiler steel [7,15,18,19].

Despite numerous studies on the heat treatment of P91 steel, the majority of studies have focused on steels in the as-received condition or on welded joints subjected to post-weld heat treatment (PWHT), particularly with regard to microstructural evolution, creep resistance, and type IV cracking behavior [11,12,13,20,21]. Limited attention has been devoted to post-operation P91 base material after long-term high-temperature service exposure, especially regarding the combined effect of normalization and different tempering variants on microstructure stability, fracture morphology, and mechanical performance. Furthermore, comparative analyses of single- and double-step tempering applied to post-service P91 steel remain insufficiently described in the literature. Therefore, the present study addresses this research gap by investigating the effect of normalization and different tempering conditions on the microstructure, fractographic characteristics, hardness, and tensile properties of post-operation P91 steel. The scientific contribution of this work lies in identifying the heat treatment conditions that restore the balance between strength and ductility while maintaining a stable microstructure suitable for further high-temperature service. In this study, the mechanical and microstructural characteristics of post-operation P91 steel (X10CrWMoVNb9-1) before and after heat treatment were examined. Static tensile tests were performed to determine the tensile strength and yield strength. Elongation to failure was determined, and hardness measurements were performed for samples subjected to different heat treatment conditions. A fractographic analysis of fracture surfaces and detailed microstructural observations were conducted using scanning electron microscopy (SEM). Metallographic studies were conducted using light microscopy (LM). The obtained results allowed the assessment of the changes occurring in the heat-treated material and enabled the identification of the tempering conditions that led to a favorable combination of strength and ductility of P91 steel, considering the material’s intended use.

## 2. Materials and Methods

In this study, the investigated material was post-operation P91 (X10CrWMoVNb9-1) steel in the form of a profile, which was used as a component of a third-stage steam superheater of an OFz425 boiler. The service time of the superheater was 1737 h. The operating temperature of the component was 560 °C. The chemical composition of the steel, confirmed by SEM/EDS analysis, is presented in Table 1. The results of the chemical composition analysis are consistent with the requirements of the standard [22].

**Figure 1:**
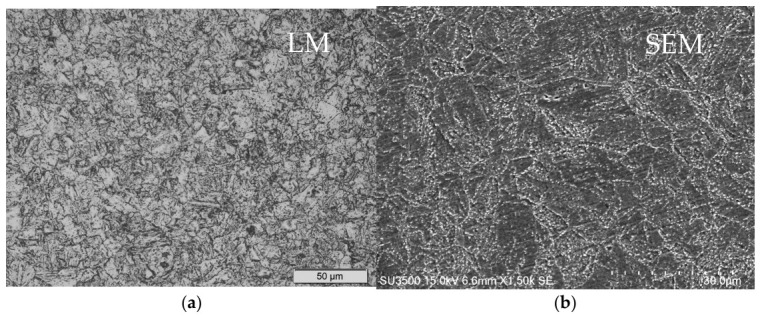
Microstructure of post-operation P91 steel in as-received condition determined (**a**) using light microscopy (LM); (**b**) using scanning electron microscopy (SEM).

The microstructure of the investigated steel in the as-received condition is shown in Figure 1. At this stage of operation, the material did not exhibit the significant degradation typical of long-term P91 steel operation. No microcracks were detected. The microstructure exhibited a characteristic lath-like arrangement of martensite, which formed irregular martensitic packets. Carbide precipitates were clearly visible as darker particles located along grain boundaries.

**Figure 2:**
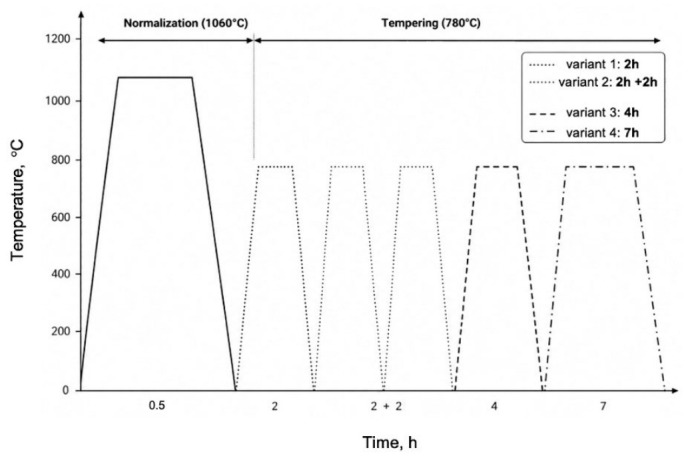
Heat treatment scheme for P91 steel: selected variants.

The samples for heat treatment were cut from the investigated material provided in the form of a rectangular profile. Normalization was performed at 1060 °C/30 min/air cooling and single- and double-stage tempering below the Ac1 temperature, i.e., at 780 °C for various durations of air cooling: 2 h, 4 h, 7 h. The heat treatment was performed in a laboratory FCF22HM chamber furnace under an air atmosphere. The samples were introduced into the furnace after the temperature had stabilized at the required level. Before heat treatment, the samples were mechanically cleaned of contaminants and surface oxides to ensure uniform heating and reduce surface oxidation. The selected heat treatments are presented in Figure 2.

**Figure 3:**
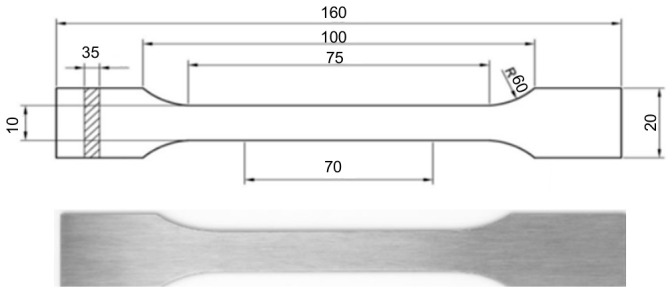
A schematic diagram of a tensile test sample and a photo of a rectangular specimen; specimen dimensions are given in mm.

Static tensile testing was performed with an INSTRON 5982 tensile testing machine (Norwood, MA, USA). For each heat treatment condition, three specimens were tested, and the mechanical properties are reported as mean values with standard deviations. Rectangular tensile specimens were used (Figure 3). The measuring length was 70 mm. The crosshead speed during the tests was 1 mm/min. During the tests, the stress–strain curves were recorded in the following system: stress σ—strain ε. Based on the force characteristics and specimen geometry measured before and after deformation, the following mechanical properties were determined: tensile strength R_m_, yield strength R_p0.2_, and elongation A.

The hardness of the tested steel was measured using the Vickers method under a load of 5 N on a longitudinal section using a 401MVD hardness tester (Instron, Norwood, MA, USA) for samples in the initial condition and after selected heat treatments. The load holding time was 10 s.

Fractographic studies of the fractures were performed using scanning electron microscopy (SEM) with a JEOL JSM 6480 microscope (Tokyo, Japan) equipped with a tungsten filament electron gun operating at an accelerating voltage of 20 kV.

Metallographic examination of the etched samples was performed using an Olympus GX light microscope (LM) (Tokyo, Japan) with an automatic scanning stage and computer-aided digital documentation using Analysis software (Tokyo, Japan, https://www.olympus-lifescience.com/, accessed 12 February 2026). Detailed microstructural observations were performed using scanning electron microscopy (SEM) and a secondary electrons detector (SE) with a JEOL JSM 6480 scanning microscope. The investigations included observations of the initial sample and the heat-treated samples. Bright-field imaging was used. The prepared material samples were embedded in a conductive resin, then mechanically ground and polished with diamond paste. To reveal the microstructure, the samples were etched by single and repeated immersion in an etching solution composed of 3.1 mL HF + 6.25 mL HNO_3_ + 25 mL H_2_O.

Quantitative microstructure analysis was performed based on images obtained using light microscopy (LM) and scanning electron microscopy (SEM). The average grain size was determined using the mean equivalent diameter method, calculated based on the surface area of individual grains. The average precipitate size was determined using an analogous procedure. The surface fraction and the distribution of precipitates within the analyzed microstructural areas were determined using image analysis methods. Image segmentation was performed based on contrast differences using consistent thresholding and image processing parameters for all analyzed heat treatment conditions, enabling differentiation between the matrix and precipitates. Quantitative analysis was performed using at least five representative microstructural images for each heat treatment condition. All analyzed SEM micrographs were obtained under identical imaging conditions and magnification. Representative microstructural areas were selected from the central regions of the samples in order to minimize edge effects and preparation-related artifacts. Grain size measurements were carried out for a minimum of 100 grains, while precipitate size and surface fraction were determined from representative analyzed regions using image analysis procedures.

## 3. Results and Discussion

### 3.1. Mechanical Properties

**Figure 4:**
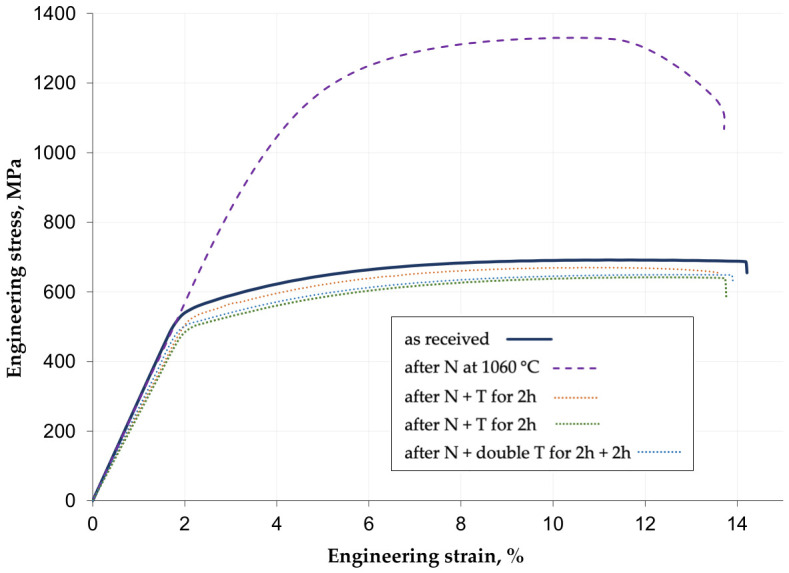
Representative stress–strain curves of P91 steel in as-received condition and after normalization and tempering.

Static tensile testing was conducted on a series of P91 steel samples in their as-received condition and after selected heat treatments. The presented stress–strain curves are representative of each heat treatment condition (Figure 4), while the average mechanical properties and corresponding standard deviations are summarized in Table 2. The tensile strength of P91 steel in its as-received condition and after operation was 692 MPa, and the yield strength reached 543 MPa. The YS/UTS ratio of 0.78 indicated retained ductility. The ultimate tensile strength and yield strength values were similar to those of P91 boiler steel in its as-received condition (before operation), according to the manufacturer’s certificate. The total elongation at break was 11.9%. The noticeable decrease in plasticity in the as-received material compared to the total elongation value of the material in its as-delivered condition is characteristic of the material after long-term operation at elevated temperatures. The sample that was heat-treated at 1060 °C for 30 min followed by air cooling exhibited an almost twofold higher ultimate tensile strength and a higher yield strength than the as-received material. At the same time, limited ductility was observed, as indicated by the total elongation to fracture of 9.9%, which is characteristic of the normalized condition. The stress–strain curve shows a clear decline after reaching the maximum stress value. The application of high-temperature tempering decreased the tensile strength to 670 MPa while increasing the material’s ductility, with the total elongation at break reaching A = 11%, indicating a partial reduction in internal stresses and partial microstructural stabilization. The yield strength was 537 MPa. Increasing the tempering time to 4 h resulted in reductions in UTS and YS values compared to those of the sample tempered for 2 h. The tensile strength and yield strength were UTS = 642 MPa and YS = 497 MPa, respectively. The YS/UTS ratio remained relatively high at 0.77, indicating stable plastic deformation capability. Double tempering (2 h + 2 h) resulted in mechanical properties similar to those obtained after 4 h of tempering. The tensile strength, yield strength, and elongation at break remained similar and were UTS = 649 MPa and YS = 511 MPa, A = 11.5%, respectively. High-temperature tempering for 7 h resulted in mechanical properties comparable to those obtained after shorter tempering times. The stress–strain curve indicates stable plastic deformation behavior with a balanced combination of strength (UTS = 649 MPa) and ductility (EL = 11.5%).

The observed decrease in strength after tempering, accompanied by improved ductility, is consistent with reports in the literature on P91 steel: tempering below the Ac1 temperature promotes recovery of the martensitic matrix and precipitation of carbides, leading to reduced hardness and tensile strength compared to the normalized condition [11,12,13,20,21]. Similar trends were observed for normalized and tempered P91 steel, in which the high strength after normalization was attributed to supersaturated martensite, whereas the subsequent tempering resulted in microstructural stabilization and improved plasticity. The differences in the absolute values of the strength and elongation between the present study and the literature data may have resulted from differences in the post-operation condition of the investigated material, prior service exposure at elevated temperature, different tempering durations, and differences in the initial microstructure.

An analysis of the stress–strain curves also provides qualitative information about the deformation behavior of the material during tensile loading. The normalized sample exhibited very high strength; however, its limited elongation and rapid stress drop after reaching the maximum stress indicated relatively low plastic deformation capability and more brittle deformation behavior. In contrast, the tempered samples exhibited a more balanced combination of strength and ductility, suggesting improved resistance to fracture and more stable plastic deformation. Among the tempered conditions, the 4 h and double-tempered variants produced a slightly higher strength–ductility balance; however, the observed differences remained relatively small. Prolonged tempering for 7 h maintained relatively high ductility; however, precipitate coarsening reduced the strengthening effect and limited further improvement in the overall mechanical response.

The mechanical properties obtained from the static tensile test were confirmed by the hardness measurements of the P91 steel in its initial state and after heat treatment (Table 2). The presented values correspond to the mean values obtained from at least three independent measurements. The highest hardness was observed in the sample after normalization at 1060 °C/30 min/air cooling (average hardness 433 HV0.5). Samples subjected to high-temperature tempering for 2 h, 4 h, and double tempering for 2 h + 2 h showed similar hardness values, significantly lower than the hardness of the samples after normalization alone: 239 HV0.5, 240 HV0.5, and 265 HV0.5, respectively. The hardness of the samples after tempering at 780 °C for 2 h, 4 h, 2 h + 2 h and air cooling was comparable to the hardness recorded for the sample in its initial, post-operation state. However, a longer tempering time, i.e., 7 h, increased the hardness of the tested steel to 303 HV0.5.

### 3.2. Fractographic Test Results

**Figure 5:**
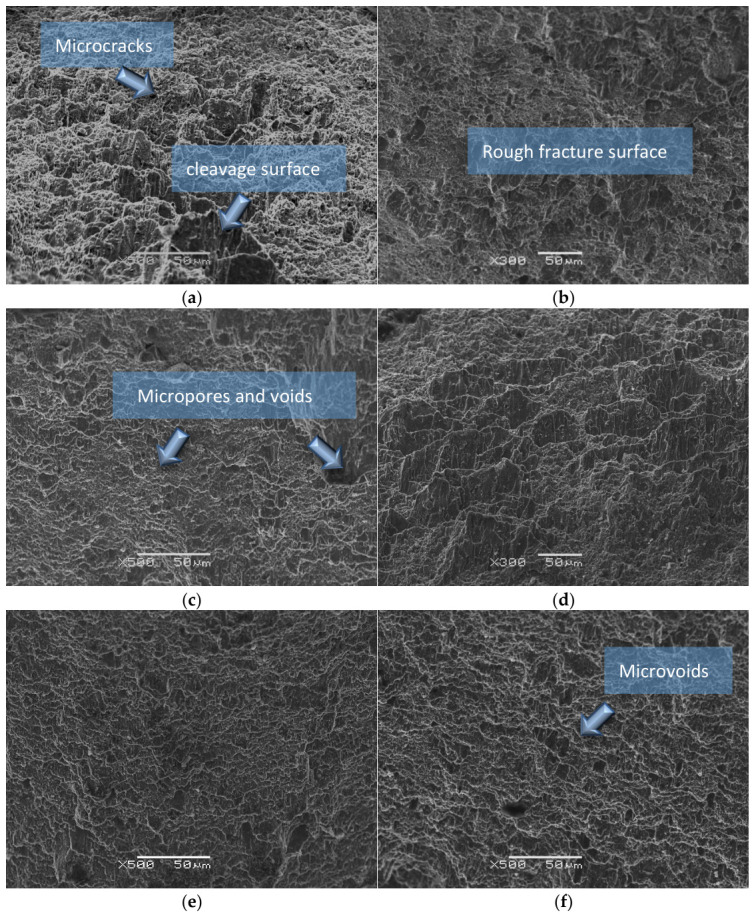
View of P91 steel fractures after static tensile test: (**a**) as-received condition; (**b**) after normalization; (**c**) after normalization + tempering for 2 h; (**d**) after normalization + tempering for 4 h; (**e**) after normalization + double tempering for 2 h + 2 h; (**f**) after normalization + tempering for 7 h.

The results of the mechanical property measurements were supported by the fractographic studies of the P91 steel. The as-received sample exhibited a mixed fracture morphology (Figure 5a). The fracture surface exhibited features characteristic of both brittle and ductile fractures, which are typical of materials subjected to prolonged operation at elevated temperatures. Elongated microcracks were visible on the fracture surface, indicating the ductile nature of the fracture. Simultaneously, initial traces of microstructural degradation were observed in the form of intergranular microcracks and zones, with characteristic smooth cleavage surfaces, typical of brittle fracture. A significantly different fracture morphology was observed in the sample subjected only to normalization, without further tempering. The observed fracture was brittle, with a clearly visible cleavage structure, a rough surface, and dominant transcrystalline fracture characteristics. The presence of needle-like and schistose formations indicated a structure containing supersaturated martensite (Figure 5b). Tempering significantly influenced the fracture morphology and fracture characteristics. The sample tempered for 2 h exhibited a fracture surface with a clearly plastic structure. Numerous microvoids of varying sizes were present, formed as a result of micropore interconnection (Figure 5c). A similar fracture characteristic was observed in the sample tempered for 4 h. The visible microvoids were deeper and more regular (Figure 5d). The sample tempered twice exhibited a plastic fracture, similar to the samples tempered for 2 and 4 h. Relatively uniformly distributed microvoids were clearly visible on the fracture surface (Figure 5e). The sample tempered for 7 h also exhibits=ed a plastic fracture, with smaller microvoids visible (Figure 5f).

The transition from brittle fracture after normalization to ductile fracture after tempering is also consistent with the mechanical behavior reported for P91 steel after heat treatment [20,21]. The brittle cleavage-like morphology observed in the normalized sample can be attributed to the presence of untempered martensite and high internal stresses, whereas the ductile dimpled morphology observed after tempering reflects stress relaxation, carbide precipitation, and improved plastic deformation behavior. The minor differences in fracture morphology compared to the data in the literature may have been associated with the post-operation state of the material and the specific heat treatment parameters applied in this study. The fractographic observations in this study were qualitative in nature and were intended to support the interpretation of the mechanical behavior and microstructural evolution of the investigated material.

### 3.3. Microstructural Evolution

**Figure 6:**
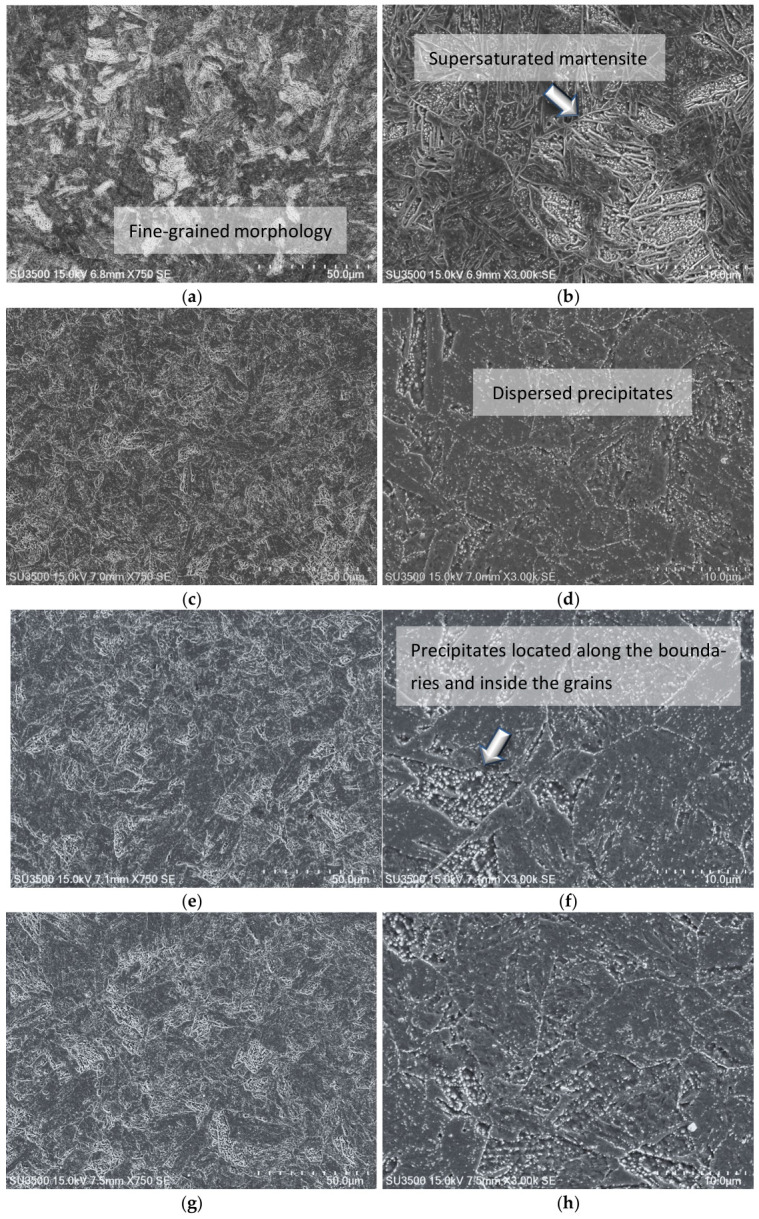

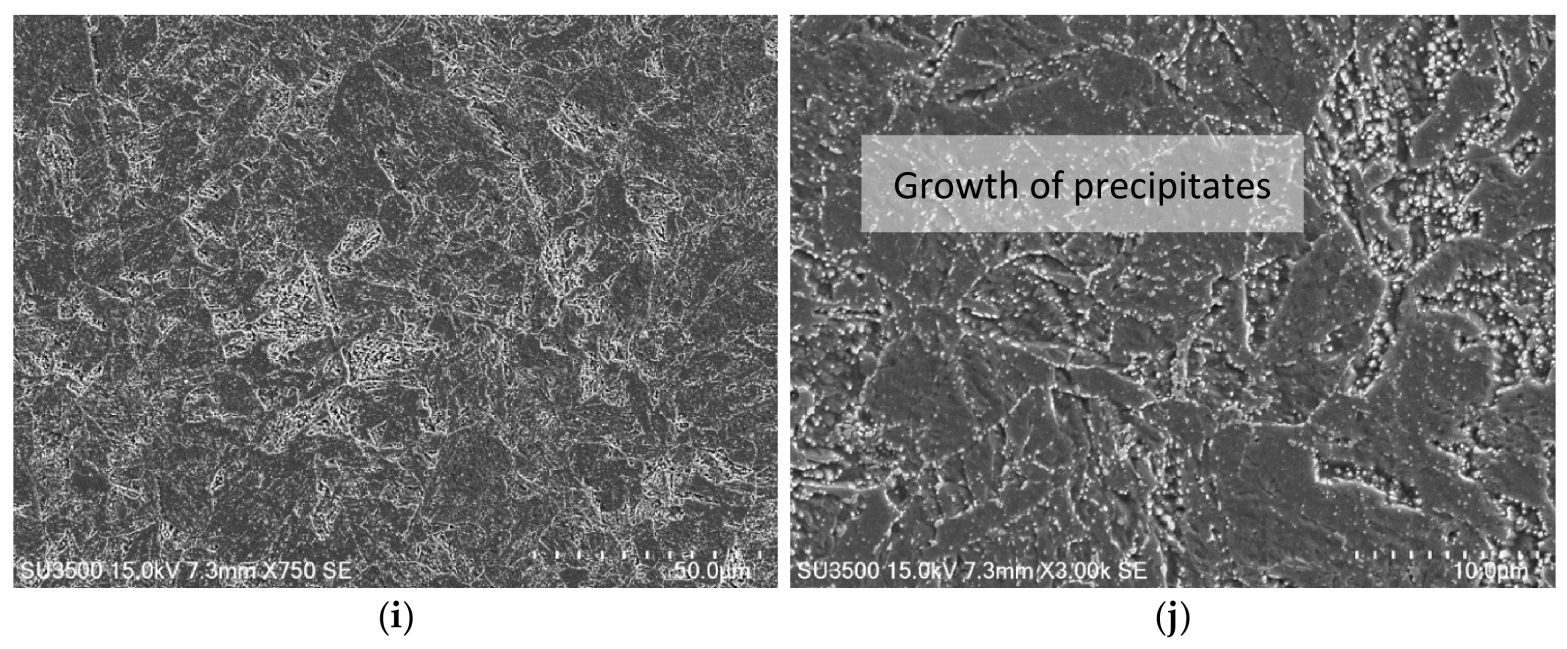
Microstructure of P91 steel as-received and after heat treatment: (**a**,**b**) after normalization; (**c**,**d**) after normalization + tempering for 2 h; (**e**,**f**) after normalization + tempering for 4 h; (**g**,**h**) after normalization + double tempering for 2 h + 2 h; (**i**,**j**) after normalization + tempering for 7 h.

Microstructure studies were performed using light microscopy (LM) and scanning electron microscopy (SEM). In the microstructure of the sample after the normalization process (without tempering), the microstructural components showed fine-grained morphology (Figure 6a,b). Supersaturated martensite was observed in the form of characteristic, sharp-edged needle-like plates. The SEM images indicate a small number of secondary precipitates. The structure of the sample tempered for 2 h was characterized by visible, well-defined grain boundaries and partially dispersed precipitates (M23C6) [20]. The martensitic structure underwent partial recovery—the needle-like martensitic plates were no longer as clearly defined as in the sample after normalization (Figure 6 c,d). This indicated the partial recovery of supersaturated martensite, the reduction in dislocation density, and the formation of dispersed carbide precipitates. The normalized condition was characterized by the presence of supersaturated lath martensite with a high dislocation density, which explained the very high strength and hardness of the material. During tempering, martensite undergoes progressive recovery and decomposition. The reduction in tetragonality, annihilation of dislocations, and precipitation of carbides contribute to the transformation of brittle untempered martensite into a more stable tempered martensitic structure. As tempering time increases, martensitic laths become less distinct and the microstructure becomes more homogeneous, resulting in improved ductility and fracture resistance. The SEM micrographs reveal numerous microprecipitates located along the grain boundaries [23,24]. In the sample tempered for 4 h, the further stabilization of the structure was observed—grain boundaries were distinct, and the martensitic morphology was reduced. Precipitates distributed along the grain boundaries and within the grains were clearly visible (Figure 6e,f). SEM observations revealed carbide precipitates forming a relatively coherent arrangement. The uniform distribution of precipitates and reduced martensitic morphology suggested effective tempering and the formation of a relatively stable tempered martensitic microstructure [21,24]. During tempering, carbon and alloying elements such as Cr, Mo, V, and Nb diffuse from the supersaturated martensitic matrix, leading to the precipitation of secondary carbides, mainly M_23_C_6_ carbides along prior austenite grain boundaries and MX-type precipitates within martensitic laths [20,21,23,24,25]. The precipitation process contributes to microstructural stabilization by reducing internal stresses and limiting recovery processes. Increasing tempering time promotes carbide coarsening and coagulation, particularly at grain boundaries, which was observed in the sample tempered for 7 h. The microstructure of the sample after double tempering was very similar to the microstructure of the sample after tempering at 780 °C/4 h/air cooling but was characterized by greater homogeneity and more-uniform precipitate distribution (Figure 6g,h). The SEM images show fine phase particles and relatively uniformly distributed carbides, which suggest the effectiveness of the double-tempering process [23,24]. In the sample tempered for 7 h, a slight growth in precipitates was observed, particularly visible along the grain boundaries (Figure 6i,j). SEM microstructure images show larger carbides (M23C6) [20,25].

The quantitative microstructure analysis (Table 3) confirms the observations presented in Figure 6a–j. In the normalized condition (without tempering), the material was characterized by the smallest average grain size of 18.0 μm and a small fraction of precipitates of 1.0%, which are consistent with the presence of supersaturated martensite with a fine, acicular morphology and a small number of secondary particles. After 2 h of tempering, the microstructure showed an increase in the proportion of secondary phases and medium precipitates, located primarily at the grain boundaries. This increase in the mean grain size indicated the initial phase of structure stabilization. Extending the tempering time to 4 h resulted in an increase in both the share of precipitates and their dispersion, which were reflected in a more uniform particle distribution in the SEM images. The double-tempered sample exhibited quantitative parameter values similar to those obtained after 4 h of tempering—an average grain diameter of 30 um and a precipitate surface fraction = 2.2%. The quantitative image analysis confirmed not only the changes in precipitate size but also the differences in precipitate distribution and surface fraction depending on tempering conditions. However, the more uniform distribution of precipitates indicates reduced heterogeneity of the martensitic matrix and improved microstructural uniformity, consistent with the SEM observations. For the longest tempering time of 7 h, a significant increase in the average precipitate size of 0.43 um was observed, with a simultaneous increase in the surface fraction to 3%. These results confirm the process of precipitate coagulation and growth, particularly along grain boundaries.

The effect of tempering duration was interpreted as competition between carbide precipitation, recovery of the martensitic matrix, and precipitate coarsening. After 2 h of tempering, the formation of fine secondary precipitates and the partial recovery of martensite indicated the initial stage of microstructural stabilization. Extending the tempering time to 4 h promoted the more uniform distribution of precipitates along the grain boundaries and within the matrix, contributing to the formation of a relatively stable tempered martensitic microstructure and an improved strength–ductility balance. The quantitative parameters of the double-tempered condition were similar to those of 4 h tempering, but the more uniform precipitate distribution suggested improved microstructural uniformity. In contrast, tempering for 7 h promoted precipitate coarsening, resulting in an increase in precipitate size and surface fraction, indicating carbide coarsening and partial coagulation, especially at grain boundaries. This reduced the effectiveness of precipitation strengthening and suggested that excessive tempering limited further improvement in mechanical performance.

The microstructural changes observed in this study are consistent with previous studies on P91 steel, which reported that tempering contributes to the decomposition of supersaturated martensite and carbide precipitation, mainly along prior austenite grain boundaries and martensitic lath boundaries [20,21,23,24,25,26,27]. The increases in precipitate size and surface fraction with increasing tempering time observed in the present work are consistent with the tendency toward carbide coarsening described in the literature. However, compared to studies performed on as-received or welded P91 steel, the present results should be interpreted in the context of post-operation material conditions, where prior service exposure could affect the initial precipitate distribution, dislocation density, and response to subsequent heat treatment.

The observed relationship between microstructural evolution and mechanical properties indicates that the mechanical behavior of the investigated P91 steel are governed by several strengthening mechanisms acting simultaneously. In the normalized condition, the dominant strengthening mechanisms are grain refinement, high dislocation density, and the presence of supersaturated martensite, resulting in very high strength and hardness. Tempering reduces dislocation strengthening due to recovery processes, while precipitation strengthening becomes increasingly important owing to the formation and dispersion of carbides. Fine and relatively uniformly distributed precipitates effectively hinder dislocation motion, contributing to maintenance of relatively high strength after tempering. However, prolonged tempering for 7 h results in precipitate coarsening, which reduces the effectiveness of precipitation strengthening and contributes to material softening.

The normalized state, characterized by fine martensite and a low fraction of precipitates ($f_{p}$ = 1%), corresponds to the highest strength (UTS = 1330 MPa), with limited ductility. As a result of tempering, increases in the fraction and distribution of precipitates ($f_{p}$ = 1.5–2.2%; ${\bar{d}}_{p}$ = 0.21–0.30 μm) are associated with a decrease in strength (UTS = 631 MPa after double tempering) and a simultaneous improvement in ductility (EL = 11.6%), which is associated with precipitate distribution, reduced martensitic morphology, and partial recovery of dislocation structure. Extending the tempering time to 7 h results in the coagulation of precipitates (${\bar{d}}_{p}$ = 0.43 μm; $f_{p}$ = 3.0%), which reduces the strengthening effect. The obtained results indicate that the characteristics of secondary precipitates significantly influence the development of the mechanical properties and the high-temperature performance of P91 steel.

## 4. Conclusions

This study investigated the effect of high-temperature heat treatment on the microstructure and mechanical properties of post-operation P91 steel (X10CrMoVNb9-1) used in steam superheater components. The influences of normalization and different tempering durations on tensile strength, yield strength, elongation, hardness, fracture morphology, and microstructural evolution were analyzed. The obtained results enabled the identification of the relationships among tempering duration, carbide precipitation behavior, martensite recovery, and the resulting mechanical performance of the investigated steel. Unlike many previous studies focused on as-received or welded P91 steel, the present work involved a comparative analysis of different tempering conditions applied to post-service material after long-term high-temperature operation. The scientific contribution of this study lies in identifying the relationships among controlled tempering, precipitate dispersion, and tempered martensitic microstructure evolution as well as the resulting balance between strength and ductility in post-operation P91 steel intended for further high-temperature service. The obtained results lead to the following conclusions:

1. P91 steel (X10CrWMoVNb9-1) in the post-operation condition exhibits strength properties similar to those of the material in the as-received condition (before operation), according to the manufacturer’s certificate, i.e., R_m_ = 692 MPa, R_0.2_ = 543 MPa. A noticeable decrease in ductility in the post-operation material (A = 11.9%) compared to the total elongation value of the material in the as-received condition (according to the manufacturer’s certificate, A = 23%) is characteristic of the material when exposed to long-term operation at elevated temperatures.
2. Normalization at 1060 °C for 30 min with air cooling led to an almost twofold increase in tensile strength (R_m_ = 1330 MPa) and a significant increase in yield strength (R_p0.2_ = 992 MPa), with a simultaneous further decrease in plasticity (A = 9.9%), which indicated the formation of a hard and brittle microstructure.
3. The use of a high-temperature tempering (780 °C) contributed to a reduction in tensile strength (R_m_ = 649 MPa after a tempering time of 7 h), while maintaining useful ductility (A = 11.5%). This suggests the effectiveness of the tempering process in reducing stresses and stabilizing the tempered martensitic microstructure.
4. A balanced combination of tensile strength (UTS = 631–670 MPa), yield strength (YS = 491–537 MPa), elongation (EL = 11.0–11.6%), and hardness (239–265 HV0.5) was obtained for samples tempered for 2–4 h and after double tempering. These tempering conditions resulted in comparable mechanical responses and tempered martensitic microstructures characterized by relatively uniform precipitate distribution, associated with a balanced strength–ductility relationship after heat treatment.
5. The hardness of the samples correlated with the tensile test results: the highest hardness (433 HV0.5) was obtained after normalization, while tempering led to a significant reduction in hardness (to the range of 239–303 HV0.5).
6. The fractographic studies indicated predominantly ductile fracture features of the samples tempered for 4 h and after double tempering (2 h + 2 h), including the presence of relatively uniformly distributed microvoids and cavities on the fracture surface.
7. The microstructural studies indicated that samples tempered for 4 h and after double tempering for 2 h + 2 h exhibited a less distinct tempered martensite lath structure, together with a relatively uniform distribution of precipitates along the grain boundaries and within the matrix. The observed precipitate dispersion suggested that these tempering conditions promoted the recovery of the martensitic structure and contributed to a more uniform microstructural arrangement. The quantitative analysis revealed an average grain size of 29–30 µm, a precipitate size of 0.26–0.30 µm, and a precipitate surface fraction of approximately 2%, suggesting relatively stable precipitate distribution and improved microstructural uniformity.

## Figures and Tables

**Table 1:** Chemical composition of P91 steel.

P91, X10CrWMoVNb9-1										
Chemical composition of steel, wt%										
C	Si	Ni	V	Cu	Mn	Cr	Mo	Nb	N	Fe
0.09	0.26	≤0.4	0.21	≤0.3	0.56	8.49	1.03	0.1	0.06	rest
Chemical composition of steel according to the PN_EN 10216-2 standard										
0.08–0.12	0.2–0.5	≤0.4	0.18–0.25	≤0.3	0.3–0.6	8.0–9.5	0.85–1.05	0.06–0.1	0.03–0.06	rest

**Table 2:** Mechanical properties of P91 steel as-received and after heat treatment; mechanical properties summarized in table are presented as mean values obtained from at least three independent measurements.

Condition	UTS, MPa	YS, MPa	YS/UTS	EL, %	HV0.5
As-received	692 ± 8	543 ± 8	0.78	11.9 ± 1.1	254 ± 5
After N ^1.^ at 1060 °C	1330 ± 14	992 ± 5	0.75	9.9 ± 0.9	433 ± 16
After N + T ^2.^ for 2 h	670 ±10	537 ± 11	0.8	11.0 ± 1.0	239 ± 9
After N + T for 4 h	642 ± 7	497 ± 7	0.77	11.2 ± 0.8	240 ± 12
After N + double T for 2 h + 2 h	631 ± 11	491 ± 10	0.78	11.6 ± 0.6	265 ± 14
After N + T for 7 h	649 ± 5	511 ± 14	0.79	11.5 ± 0.3	303 ± 20

UTS, ultimate tensile strength; YS, yield strength; EL, total elongation to failure; HV, Vickers hardness. ^1.^ N, normalization; ^2.^ T, tempering.

**Table 3:** Microstructural parameters of P91 steel after heat treatment.

Condition	$\bar{d}$, [μm]	${\bar{d}}_{p}$, [μm]	$f_{p}$, [%]
As-received	28.0	0.25	3.0
After N ^1.^ at 1060 °C	18.0	0.14	1.0
After N + T ^2.^ for 2 h	26.0	0.21	1.5
After N + T for 4 h	29.0	0.26	2.0
After N + double T for 2 h + 2 h	30.0	0.30	2.2
After N + T for 7 h	34.0	0.43	3.0

^1.^ N, normalization; ^2.^ T, tempering; $\bar{d}$, average grain size; ${\bar{d}}_{p}$, average size of precipitates; $f_{p}$, precipitate surface fraction.

## Data Availability

The original contributions presented in the study are included in the article, further inquiries can be directed to the corresponding author.

## Abbreviations

SEM	scanning electron microscopy
LM	light microscopy
UTS	ultimate tensile strength
YS	yield strength
EL	total elongation
N	normalization
T	tempering
